# HSF5: A novel switch for meiotic pachynema progression in male germ cells

**DOI:** 10.1016/j.gendis.2025.101868

**Published:** 2025-09-23

**Authors:** Chunhai Luo, Ziqi Yu, Dalin Liu, Haoran Xu, Junfeng Zhan, Fei Sun

**Affiliations:** Department of Urology & Andrology, Sir Run Run Shaw Hospital, Zhejiang University School of Medicine, Hangzhou, Zhejiang 310016, China

Meiosis, a specialized cell division process that generates haploid gametes for sexual reproduction, includes the crucial phase of prophase I, where homologous chromosomes pair, synapse, and undergo recombination. Prophase I is divided into five stages—leptonema, zygonema, pachynema, diplonema, and diakinesis—with pachynema, lasting approximately six days in mice, being the longest and playing a uniquely critical role in meiosis. The regulatory mechanisms driving pachynema progression are intricate and not yet fully understood, though the pachytene checkpoint is most frequently implicated as crucial for ensuring proper meiotic progression. Additionally, three key meiotic checkpoints play critical roles in maintaining the fidelity of pachynema progression: the DNA damage checkpoint, which acts as a recombination-dependent arrest mechanism, triggering apoptosis if double-strand breaks (DSBs) are not fully repaired during pachynema; the synapsis checkpoint, which ensures accurate chromosome pairing and synapsis, eliminating meiocytes with asynapsis or synaptic errors; and meiotic sex chromosome inactivation (MSCI), which mediates the transcriptional repression of unsynapsed regions in the sex chromosomes within the XY body, with defective MSCI leading to complete meiotic arrest and elimination of spermatocytes at the mid-pachytene stage.^1^ However, several studies have suggested that non-checkpoint mechanisms may also play important roles in pachynema progression. For instance, while the underlying molecular mechanisms remain unclear, the absence of proteins such as HSPA2 and EIF4G3 leads to meiotic arrest at the pachytene stage, despite no significant cytological defects in DSB repair, synapsis, or MSCI.^2^ These findings raise the possibility that, in addition to the pachytene checkpoint, which monitors and ensures proper recombination, synapsis, and MSCI, other distinct regulation mechanisms for pachynema progression may also exist.

Is there a master regulator or switch for meiotic pachynema progression in male germ cells? A recent study published in *Nucleic Acids Research* delves into this question, uncovering a novel switch for meiotic pachynema progression.^3^ This switch may function independently of or in concert with the classical pachytene checkpoint, acting as a sentinel to facilitate further progression through pachynema and ensuring the proper continuation of meiosis. The authors identified a non-canonical member of the heat shock factor family (HSF5) as a chromatin-associated protein specifically expressed in germ cells during early to mid-pachytene spermatocytes, persisting until step 4 of round spermatids, after which its expression declined. *Hsf5* knockout (*Hsf5* KO) mice exhibited impaired pachynema progression, increased spermatocyte apoptosis, and male infertility. Although HSF5 loss caused meiotic arrest at the mid-to-late pachytene stage, it did not significantly affect pachytene checkpoint events (DSB repair, synapsis, recombination, and MSCI), and HSF5-deficient spermatocytes were still able to condense metaphase I chromosomes, as demonstrated by the okadaic acid induction experiment. This suggests that HSF5 likely functions as a switch for pachynema progression, driving spermatocytes transition from a pachytene checkpoint-passed state into the next phase of meiosis and ensuring proper exit from pachynema (Fig. 1). Specifically, in wild-type pachytene spermatocytes, HSF5 coordinates with chromatin remodeling complex components (*e.g.*, SMARCA5, SMARCA4, SMARCE1) to mediate the progressive down-regulation of synaptonemal complex and meiosis-specific genes (*Sycp1*, *Meiob*, *Msh4*) during the transition from early to late pachytene. Concurrently, HSF5 facilitates the transcriptional activation of cell cycle regulators (*Hspa2*, *Ccnb1*, *Plk1*) critical for meiotic progression. HSF5 deficiency disrupts this regulatory cascade, resulting in aberrantly sustained transcription of genes that are normally silenced during late pachytene, and insufficient activation of genes required for meiotic advancement. Consequently, spermatocytes fail to exit pachytene, leading to meiotic arrest and disrupted spermatogenesis (Fig. 1).

**Figure 1:**
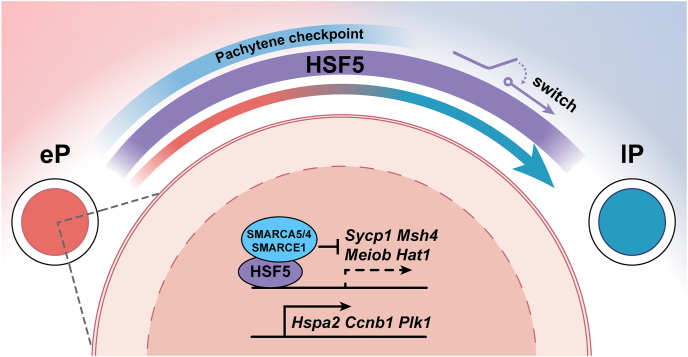
The schematic diagram illustrating the proposed function of HSF5 as a regulatory switch controlling pachynema progression in male meiosis.

However, it remains unclear how HSF5 differentiates between autosomal and sex chromosomal regions during its chromatin-binding activities. The protein's predominant localization in the XY body suggests a possible role in MSCI, though this hypothesis is not strongly supported by existing findings that HSF5 does not interact strongly with sex chromosome-specific factors (*e.g.*, BRCA1, ATR) or employs distinct binding motifs between autosomal and sex chromosomal regions. Besides, while pachynema arrest in *Hsf5* KO mice has been well characterized, the downstream effects on post-meiotic events, such as histone-to-protamine transition, sperm chromatin compaction, and post-meiotic sex chromatin (PMSC) structure formation, were not fully explored. This is particularly relevant since the transcriptional suppression initiated by MSCI extends beyond meiosis I, continuing into meiosis II and spermiogenesis, well after the dissolution of the XY body. Moreover, HSPA2 functions as a molecular chaperone for CDC2 in the mouse testis, promoting the formation of the CDC2/cyclin B1 complex essential for initiating the G2/M-phase transition in meiotic cell cycles. HSF5 preferentially binds to the HSPA2 promoter regions, and *Hspa2* expression is significantly reduced upon disruption of HSF5. Based on these observations, can it be hypothesized that HSF5 acts as a master regulator of *Hspa2* transcription, together playing a critical role in initiating synapsis and promoting pachytene exit?

This study provides groundbreaking insight into the molecular dynamics of pachynema progression, identifying HSF5 as a novel regulatory switch at the forefront of this process. These findings could also offer valuable insights into the diagnosis and treatment of male infertility. For instance, HSF5 may serve as a diagnostic marker for clinical cases of idiopathic infertility, such as those seen in non-obstructive azoospermia patients, and could provide a foundation for the development of gene-editing therapies for male infertility in the future.

## Funding

This work was supported by grants from The National Natural Science Foundation of China (No. U24A20657, 82371613 to F.S), The Key Research and Development Program of Zhejiang Province (No. 2023C03035 to F.S), National Natural Science Foundation of China [82301798], China Post-doctoral Science Foundation [2022M722767], and Fellowship of China National Postdoctoral Program for Innovative Talants [BX20230314].

## CRediT authorship contribution statement

**Chunhai Luo:** Writing – original draft, Writing – review & editing, Conceptualization. **Ziqi Yu:** Visualization. **Dalin Liu:** Validation. **Haoran Xu:** Visualization. **Junfeng Zhan:** Writing – review & editing, Funding acquisition. **Fei Sun:** Funding acquisition.

## Conflict of interests

The authors have no competing interests to declare.
